# Establishment of Novel High-Standard Chemiluminescent Assay for NTPase in Two Protozoans and Its High-Throughput Screening

**DOI:** 10.3390/md18030161

**Published:** 2020-03-13

**Authors:** Masamitsu Harada, Jun Nagai, Riho Kurata, Kenji Shimizu, Xiaofeng Cui, Takayuki Isagawa, Hiroaki Semba, Jun Ishihara, Yasuhiro Yoshida, Norihiko Takeda, Koji Maemura, Tomo Yonezawa

**Affiliations:** 1 Center for Therapeutic Innovation, Gene Research Center for Frontiers Life Sciences, Nagasaki University, Graduate School of Biomedical Sciences, 1-12-14 Sakamoto, Nagasaki 852-8523, Japan; vokuaoto@me.com (M.H.); JNAGAI@PARTNERS.ORG (J.N.); 2 Education and Research Center for Pharmaceutical Sciences, Osaka University of Pharmaceutical Sciences, 4-20-1 Nasahara, Takatsuki, Osaka 569-1094, Japan; kurata@gly.oups.ac.jp; 3 Division of Immune Regulation, Institute for Genome Research, Tokushima University, Tokushima-shi, Tokushima 770-8505, Japan; kshimizu@genome.tokushima-u.ac.jp; 4 School of Chemistry, Chemical Engineering and Life Sciences, School of Materials and Engineering, Wuhan University of Technology, 122 Loushi Rd, Wuhan 430070, Hubei, China; xfc.cui@gmail.com; 5 Department of Cardiovascular Medicine, Nagasaki University Graduate School of Biomedical Sciences, 1-7-1 Sakamoto, Nagasaki 852-8501, Japan; i-takayuki13@nagasaki-u.ac.jp (T.I.); maemura@nagasaki-u.ac.jp (K.M.); 6 Department of Cardiovascular Medicine, The Cardiovascular Institute, Tokyo Japan Nishiazabu 3-2-19, Minato-ku, Tokyo 106-0031, Japan; hiroaki_se@yahoo.co.jp; 7 Graduate School of Biomedical Sciences, Nagasaki University, 1-14 Bunkyo-machi, Nagasaki 852-8521, Japan; jishi@nagasaki-u.ac.jp; 8 Department of Immunology and Parasitology, University of Occupational and Environmental Health, 1-1 Iseigaoka, Yahatanishi-ku, Kitakyushu 807-8555, Japan; freude@med.uoeh-u.ac.jp; 9 The University of Tokyo, Department of Cardiovascular Medicine, Graduate School of Medicine, 7-3-1, Hongo, Tokyo, Bunkyo-ku 113-8654, Japan; ntakeda-tky@umin.ac.jp

**Keywords:** *Toxoplasma gondii*, *Neospora caninum*, NTPase, HTS, synthetic compound library, marine bacteria extracts

## Abstract

*Toxoplasma gondii* is a major protozoan parasite and infects human and many other warm-blooded animals. The infection leads to Toxoplasmosis, a serious issue in AIDS patients, organ transplant recipients and pregnant women. *Neospora caninum*, another type of protozoa, is closely related to *Toxoplasma gondii.* Infections of the protozoa in animals also causes serious diseases such as Encephalomyelitis and Myositis-Polyradiculitis in dogs or abortion in cows. Both *Toxoplasma gondii* and *Neospora caninum* have similar nucleoside triphosphate hydrolases (NTPase), NcNTPase and TgNTPase-I in *Neospora caninum* and *Toxoplasma gondii*, respectively. These possibly play important roles in propagation and survival. Thus, we targeted the enzymes for drug discovery and tried to establish a novel high-standard assay by a combination of original biochemical enzyme assay and fluorescent assay to determine ADP content. We then validated whether or not it can be applied to high-throughput screening (HTS). Then, it fulfilled criterion to carry out HTS in both of the enzymes. In order to identify small molecules having inhibitory effects on the protozoan enzyme, we also performed HTS using two synthetic compound libraries and an extract library derived from marine bacteria and then, identified 19 compounds and 6 extracts. Nagasaki University collected many extracts from over 18,000 marine bacteria found in local Omura bay, and continues to compile an extensive collection of synthetic compounds from numerous drug libraries established by Japanese chemists.

## 1. Introduction

*Toxoplasma gondii* is a major type of parasites and an obligatory single-cell parasitic eukaryote [1] causing serious health issues in human beings [2] and many warm-blooded animals [1]. The infection leads to Toxoplasmosis, which is particularly acute in AIDS patients [3], organ transplant recipients [4] and pregnant women [5]. *Neospora caninum* was recently discovered to be another type of protozoa belonging to phylum Apicomplexa [6]. It infects animals such as dogs and cows [6], resulting in Encephalomyelitis and Myositis-Polyradiculitis in dogs [7,8], and abortion in cows [9,10]. *Neospora caninum* also exhibits close similarities in morphology and phylogenetics of *Toxoplasma gondii* [6,11]. Indeed, these two protozoa share a novel nucleoside triphosphate hydrolase (NTPase; EC3.6.1.15), which is different from the common ecto-ATPase [11]. The protozoa contain NTPase of up to 2 to 8% of total protein, which is remarkable as dormant enzyme in its tachyzoite stage [12]. It accumulates in dense secretory granules and is secreted into the parasitophorous vacuole [13]. NTPase is activated by dithiothreitol in biochemical enzymatic validation [14]. The predicted inducers including dithiol compound, unknown dithiol-disulfide oxidoreductase or something else of that nature, have not yet been identified in the parasitic cell. The enzyme is thought to be important in releasing tachizoite from infected host cells [14].

*Toxoplasma gondii* has two NTPase isoforms, type I enzyme TgNTPase-I and type II enzyme, TgNTPase-II. Type I enzyme prefers triphosphate nucleosides as substrate and type II enzyme hydrolyzes both triphosphate and diphosphate nucleosides [15]. Both enzymes share 97% homology in amino acid levels [15]. *Neospora caninum* also has a counterpart of TgNTPase-I, which is called as NcNTPase, and its homology is 73% in its amino acid level [16]. Prof. Asai and Prof. Harada succeeded in generating recombinants of both TgNTPase-I and NcNTPase using E. Coli., and converted active mutants of both enzymes as covered in their previous report [17]. They kindly gifted the two active mutants to us. Both enzymes convert ATP to ADP in vitro, therefore we thus set out to utilize ADP content hydrolyzed by the enzyme to measure NTPase activity. We also focused on the high-standard assay to determine ADP content by fluorescence and enzymatic reaction in a previous work [18] trying to validate whether or not it can be used to monitor the activity of NTPase.

In this study, we focused on protozoan NTPase as drug targets for Toxoplasmosis in both human and animals. A novel high-standard and -dynamic-range assay was established by combining classical enzyme assay and fluorescent assay to measure ADP content. We also validated whether or not the assay can be applied to high-throughput screening (HTS). Finally, in order to identify synthetic compounds and extracts derived from marine bacteria having inhibitory effects on the NTPase, we performed several HTSs and identified 19 compounds and 6 extracts.

## 2. Results

### 2.1. Amino Acid Sequences of TgNTPase and NcNTPase and Structural Information of Their Active Mutants

**Figure 1:**
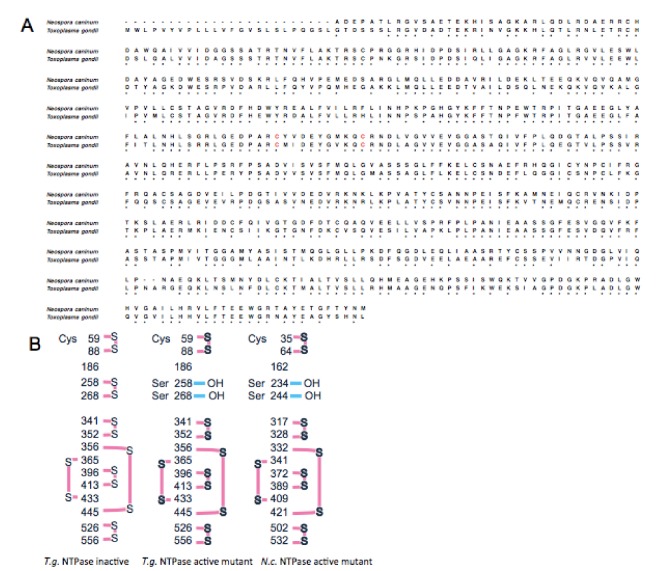
Amino acid sequences of *Toxoplasma gondii* and *Neospora caninum* NTPase and structural information of their active mutants. (**A**) Amino acid sequences of TgNTPase-I (lower raw) and NcNTPase (upper raw). Red indicates two important cysteine residues for locking enzymatic activity at 258 and 268 of TgNTPase-I, or at 234 and 244 of NcNTPase. (**B**) Structural information of disulfide-bond on TgNTPase inactive (left), TgNTPase active mutant (middle) and NcNTPase active mutant (right).

We compared a homology between TgNTPase-I and NcNTPase (Accession No. AB525222 and Q27893, respectively) and its homology is 70% in amino acid level (Figure 1A). C258 and C268 of TgNTPase-I and C234S and C244S of NcNTPase are important for locking the enzyme activity as assigned by red letter in Figure 1A. Prof. Asai and Prof. Harada established TgNTPase-I C258S, C268S and NcNTPase C234S, C244S active mutant as shown in Figure 1B. 

### 2.2. Establishment of Novel High-Standard Assay to Determine NTPase Activity by Combination of Classical Enzymatic Assay and Fluorescent Assay to Measure ADP Content

**Figure 2:**
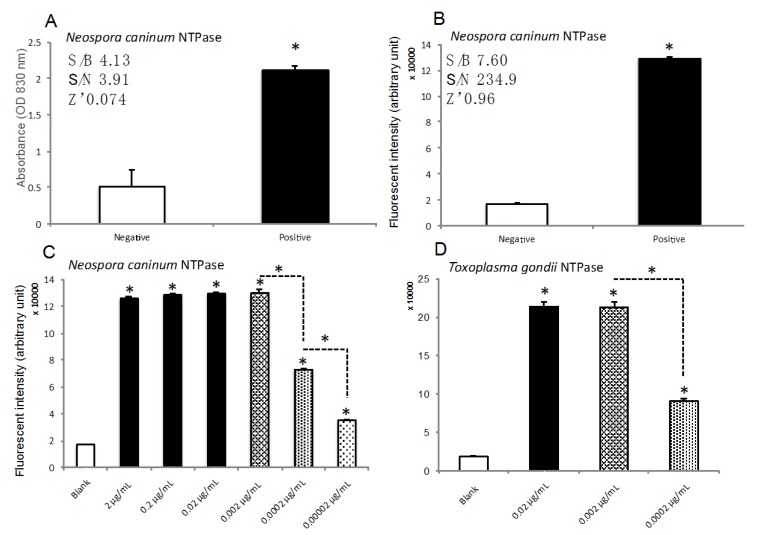
Establishment of novel high-standard assay to determine NTPase activity by combination of classical enzymatic assay and fluorescent assay to measure ADP content. Enzymatic activity of NcNTPase (0.002 μg/mL) was measured by classical absorbance assay (**A**) or novel assay combination of classical enzymatic and fluorescence assay by measuring ADP content (**B**). (**C**) The activities of various concentration of NcNTPase at 0.00002, 0.0002, 0.002, 0.02 or 2 μg/mL. (**D**) The activities of various concentration of TgNTPase at 0.0002, 0.002, 0.02 or 2 μg/mL. The data are expressed as means ± SEM (*n* = 3 or 4). Asterisk: Was considered to be statistically significant if their *P* values were *P* < 0.05.

We first checked activity of recombinant NTPase active mutant by classical enzymatic assay and observed a significant increase of NcNTPase activity compared to substrate solution without the enzyme (Figure 2A left). The S/B, S/N and Z’ were 4.13, 3.91 and 0.074. The accuracy is not adequate for carrying out HTS. Thus, we used high-sensitive ADP fluorescent assay after NTPase enzymatic reaction and succeeded in establishing a novel assay for NTPase. The sensitivity and accuracy were improved greatly as the S/B, S/N and Z’ were 7.60, 234.9 and 0.96 (Figure 2A right). Similar results were also observed in TgNTPase by both classical enzymatic assay and novel assay (Table 1). Next, we validated whether or not the assay can be applied to HTS using a robot arm. As shown in Supplementary Figure S1, we added an enzyme reaction solution with or without NTPase using the robot arm and then did the incubation at 37 degrees for 10 min. We then added 0.1N HCl to stop the enzymatic reaction. After that, we added an equivalent biofluorescent reaction solution by the roboto arm to measure ADP content. Eventually, we measured fluorescent intensities by PHERAstar FS at ex. 540 nm/em. 590 nm. In order to validate the half maximal concentration of NTPase for HTS, we prepared a serial dilution of NcNTPase ranging from 0.00002 to 2 μg/mL by a dilution rate of 1/10 and then, determined the fluorescent intensities by robot arm. We observed the concentration-dependency from 0 to 0.002 μg/mL (Figure 2C). We also obtained similar results in TgNTPase (Figure 2D and Table 1). Approximate concentration of the half maximal was 0.0002 μg/mL. We summarized the sensitivities and accuracies of several independent assays in Table 1.

### 2.3. HTS Using a Synthetic Compound Library, Which is Provided by the University of Tokyo, in Order to Identify Compounds Inhibiting NcNTPase

**Figure 3:**
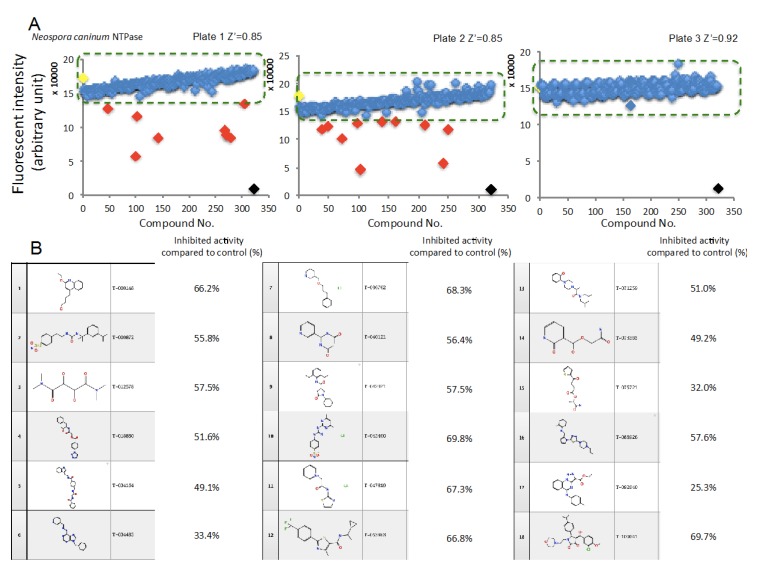
High-throughput screening (HTS) using a synthetic compound library, which is provided by the University of Tokyo, in order to identify compounds inhibiting NcNTPase. (**A**) The results of HTSs. Green dashed line indicates threshold as average of positive control ± 30% of its activity. Red rhombus indicates less than the threshold. The data of positive control (Yellow rhombus, 0.002 μg/mL NcNTPase) or negative control (Black rhombus, 0.5% DMSO) are expressed as means ± SEM (*n* = 16). (**B**) Structural information and inhibited activities of hit compounds.

Having succeeded in establishing a high-standard assay for NTPase, we decided to carry out a pilot HTS to identify compounds inhibiting NcNTPase. We used a part of the synthetic compound library named ‘Core library 9600’, which is consisted of established drug-like compounds and similar structure of these and provided by the University of Tokyo, at 10 μM and then, identified 22 from 960 compounds using threshold set as average of positive control—3× SD (Figure 3A). All of the assays had very good qualities to carry out HTS (Figure 3A). Among these, compounds, which had more than 30% inhibition, were assigned as ‘Hit’. Eighteen Hit compounds and their inhibited enzymatic activities normalized to 0.5%DMSO were shown in Figure 3B.

### 2.4. HTS Using a Synthetic Compound Library, Which is Provided by Nagasaki University, in Order to Identify Compounds Inhibiting TgNTPase

**Figure 4:**
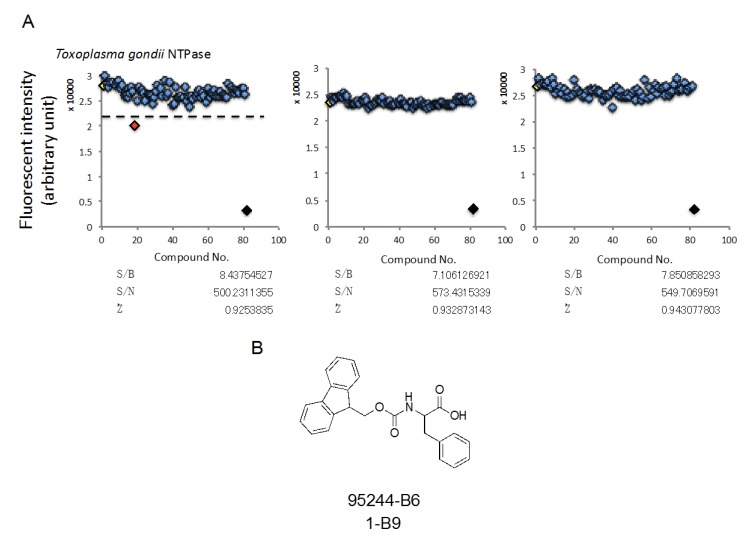
HTS using a synthetic compound library, which is provided by Nagasaki University, in order to identify compounds inhibiting TgNTPase. (**A**) The results of HTSs. Dashed line indicates threshold as average of positive control—3× SD. Red rhombus indicates less than the threshold. The data of positive control (Yellow rhombus, 0.002 μg/mL TgNTPase) or negative control (Black rhombus, 0.5% DMSO) are expressed as means ± SEM (*n* = 16). (**B**) Structural information and inhibited activities of hit compounds.

In order to identify novel structural compounds, we used a synthetic compound library, which was synthesized by many Japanese chemists, and then, performed HTS. We used 270 synthetic compounds at 10 μM and then, identified one compound using threshold set as average of positive control—3× SD (Figure 4A upper). All of the assays were fulfilled the criterion to carry out HTS (Figure 4A lower). The hit compound is described in Figure 4B. It had 30% inhibition rate compared to 0.5%DMSO. 

### 2.5. HTS Using an Extract Library Derived from Marine Bacteria in Order to Identify Extracts Inhibiting TgNTPase

**Figure 5:**
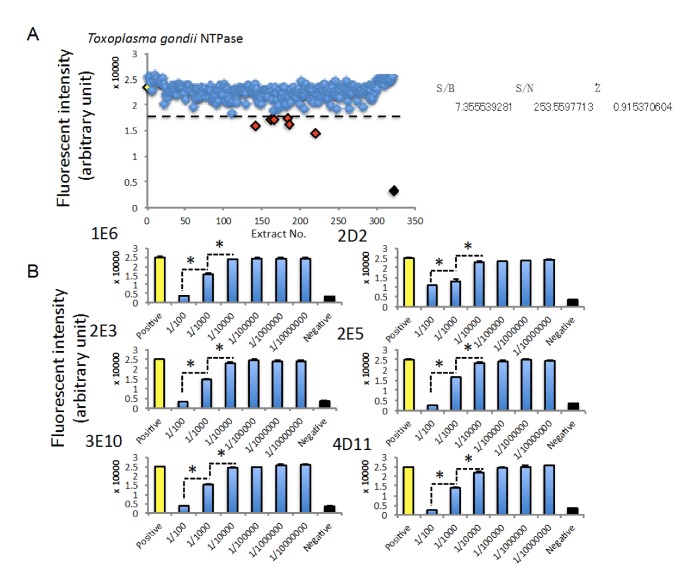
HTS using an extract library derived from marine bacteria in order to identify extracts inhibiting TgNTPase. (**A**) The results of HTSs. Dashed line indicates threshold as average of positive control—3× SD. Red rhombus indicates less than the threshold. The data from positive control (Yellow rhombus, 0.002 μg/mL TgNTPase) or negative control (Black rhombus, 0.5% DMSO) are expressed as means ± SEM (*n* = 16). (**B**) Extracts identified by 1st HTS inhibited TgNTPase activity in a concentration-dependent manner. The data are expressed as means ± SEM (*n* = 8 or 16). Asterisk: Was considered to be statistically significant if their *P* values were *P* < 0.05.

Finally, we tried to perform HTS using a natural extract library extracted from marine bacteria. To identify extracts possessing inhibitory effects on TgNTPase, we performed HTS using 320 extracts derived from marine bacteria and then, identified 6 extracts, which had more than 25% inhibition compared to 0.5% DMSO (Figure 5A). The assay qualities also fulfilled with criterion to carry out HTS (Figure 5A). We also observed that all of the 6 extracts inhibit TgNTPase activity in a concentration-dependent manner (Figure 5B). 

## 3. Discussion

In this study, we for the first time established a novel high-accuracy and -dynamic-range assay for measuring protozoan NTPase using a combination of classical enzymatic assay and fluorescent assay to determine ADP content as previously established [18]. From what we have observed, the assay has a very good dynamic-range, around 8-fold normalized to blank and its Z’ values are usually more than 0.8 and thus, can carry out HTS for drug discovery. We demonstrated that even in two different protozoa enzymes, the assay similarly works to reflect the enzymatic activities. Contrary, classical enzymatic assay has a poor-dynamic-range and -accuracy. Additionally, it would be convenient and an easy process if the fluorescent assay to determine ADP is combined to the classical enzymatic assay by simply adding the equivalent biofluorescent reaction solution to samples after stopping the enzymatic reaction. However, it would be important to simultaneously add reagents to all of the samples using a robot arm. Indeed, we accomplished three independent HTSs and identified 19 synthetic compounds and 6 extracts derived from marine bacteria. We also identified a phenylalanine derivative from 240 compounds, which are a part of synthetic compound library provided by Japanese chemists working at the leading institutions. Additionally, we identified 18 synthetic compounds from 960 compounds, which are part of the Core library provided by a Drug Discovery Initiative at the University of Tokyo. T-34483 and T-092040, from among the hit compounds, possess a remarkable inhibitory effect on NTPase. These have similar chemical structures to 2 phenylthiol-indole, and coincidently mirror a previous report by Asai et al. [19]. In the near future we will validate more derivatives and examine the structure-activity relationship with other structural compounds. We could not establish a valid counter-screen assay for NTPase because two enzymes in both protozoa are too close. However, less specificity of the compound may be effective against both protozoan enzymes even if in double infection. We will of course validate the specificity of the hit compound among several relative enzymes in future.

Eventually, we set out to perform HTS using 320 extracts from marine bacteria in order to identify an antagonist of TgNTPase. Our University collaborated with Prof. Yamada and will continue working toward establishing a novel drug library using our 18,000 plus extracts from marine bacteria. We will follow up our success in identifying 6 extracts inhibiting NTPase activity in a concentration-dependent manner with fractionating the extract and identify a single ingredient affecting it.

From the viewpoint of clinical diagnostics, both NTPase I and II in *Toxoplasma gondii* were shown to use a feasible blood marker in acute toxoplasmosis patients, and its diagnostic rate was 93% by ELISA, mirroring Sabin-Feldman dye test titer, a well-known serological test for toxoplasmosis [20]. Although we do not know whether or not active NTPase is in the blood of infected subject, the high-sensitive assay for NTPase in this study may be used for clinical diagnostics of toxoplasmosis.

In conclusion, we, for the first time, established a novel high-standard assay for protozoan NTPase and demonstrated several series of HTS, through the process of identifying 6 extracts and 19 compounds from extract and synthetic compound libraries. It is an indispensable tool for developing medicaments for protozoa infections.

## 4. Materials and Methods

### 4.1. Materials

TrisHCl, Glucose, H_2_SO_4_, HCl, Resazurin, BSA, N-ethylmaleimide, HEPES, Mg(CH_3_COO)_2_ and (NH_4_)_6_Mo_7_O_24_·4H_2_O were obtained from Wako Pure Chemical Industries, Ltd., Chuo-ku, Osaka, Japan. ATP, G6P dehydrogenase and NADP were obtained from Oriental Yeast, Co, Ltd., Itabashi-ku, Tokyo, Japan. ADP-hexokinase was obtained from Asahi Kasei Pharma Co., Ltd., Chiyoda-ku, Tokyo, Japan. Diaphorase-I was obtained from Unitika Ltd., Chuo-ku, Tokyo, Japan. Fiske-Subbarow Reducer was obtained from Sigma Aldrich, St. Louis, MO, USA. Triton X-100 and DMSO were obtained from Nacalai Tesch, Inc., Kyoto, Japan. Both TgNTPase-I and NcNTPase active mutant were kindly gifted from Prof. Asai and Prof. Harada. Extracts from marine bacteria and synthetic compound library were kindly gifted from Prof. Takeda and Prof. Ishihara, respectively. Core library 9600 was kindly provided by Drug Discovery Initiative in the University of Tokyo. Core libarary 9600 mainly consisted of compounds having similar structures of established drugs. Nagasaki University has kept collecting many extracts from over 18,000 marine bacteria in our local sea around Omura bay and prepared their extracts as a drug library. Prof. Hatakeyama and Prof. Ishihara’s group have kept gathering synthetic compounds including a novel structure provided from many Japanese chemists at Universities or Institutes and provided these as a drug library.

### 4.2. Preparation of Extracts from Marine Bacteria

Marine bacteria were inoculated on plates made by Marine Agar 2216 (Difco Laboratories, Detroit, MI, USA) at 26 degrees overnight and then transferred to 2 mL Marine Broth 2216 (Difco Laboratories, Detroit, MI, USA) at 26 degrees overnight. Preculture turbid broth was transferred to 200 mL Marine Broth and incubated at 26 degrees overnight. After incubation, 60 mL acetone was added to the broth. The suspensions were sonicated for 5 min in water bath. The suspension and the filtrate was filtered under reduced pressure and transferred to evaporation flask, respectively. The suspensions were mixed for 3 min on a shaker at 230 rpm. The mixture was separated into organic and aqueous layer and the aqueous layer was drained. Ethyl acetate was evaporated under reduced pressure at 20 hPa at 40 degrees and the residual ethyl acetate was removed under reduced pressure at 1 hPa overnight. The crystal solids inside the evaporation flask was dissolved in DMSO to give a concentration of 100 mg/mL. the sample was kept at −80 degrees until the experiments. We only prepared marine bacteria, which can be grown on the plate made by Marine Agar 2216, and have not yet determined their species completely. At least, these includes gram negative *Bacilii*, actinomycetes and so on.

### 4.3. Classical Enzymatic Assay for NTPase

We compared a homology between TgNTPase-I and NcNTPase (Accession No. AB525222 and Q27893, respectively.) and its homology is 70% in amino acid level (Figure 1A). C258 and C268 of TgNTPase-I and C234S and C244S of NcNTPase are important for locking the enzyme activity as assigned by red letter in Figure 1A. Prof. Asai and Prof. Harada established TgNTPase-I C258S, C268S and NcNTPase C234S, C244S active mutant as shown in Figure 1B. Basically, the assay was done according to previous report [19]. In brief, 2.5% MoAm in 5N H_2_SO_4_, Recombinant and Fiske-Subbarow Reducer solution, which is 0.16 g in 1mL DDW, were mixed at ratio of 4:1 as colorimetric solution. NTPase at indicated concentration were dissolved in HEPES-KOH (pH 7.5) buffer containing 1 mM ATP and 6 mM Mg (CH_3_COO)_2_ and then, incubated at 37 °C for 10 min. After incubation, 0.1 N HCl at the half volume of enzymatic reaction solution was added to stop enzymatic reaction and then the same volume of the colorimetric solution was added for developing. Ten min after that, absorbance at 830 nm was measured.

### 4.4. Combination Classical Enzymatic Assay with Fluorescent Assay for ADP

We prepared 2× biofluorescent reaction solution, which is 100 mM Tris-HCl (pH 7.5) containing 10 mM MgCl_2_, 2 mM glucose, 2 U/mL ADPHK, 2 U/mL G6PDH, 2U/mL Diaphorase I, 200 μM NADP, 100 μM Resazurin in DMSO, 0.01% BSA, 0.05% Triton X-100 and 20 mM N-ethylmaleimide in DMSO according to a previous report [18]. We performed enzymatic reaction according to 4.2 in this section. After stopping enzymatic reaction, we added equal amount of 2× biofluorescent reaction solution to enzymatic reaction solution and then, measured fluorescence intensity at ex. 540/em. 590 nm by PHERAstar FS (BMG LABTECH JAPAN L.t.d., Saitama, Japan).

### 4.5. HTS Using Robot Arm

As shown in Supplementary Figure S1, initially, vehicle, 0.5 μL synthetic compounds or extracts (final 0.05% DMSO) were put onto 384 well-plate, and using a robot arm (12 stage-workstation EDR-384 SX, Biotech Co., Ltd., Bunkyu-ku, Tokyo, Japan), all wells were injected, except for negative control, with 4.5 μL of enzymatic reaction solution containing 0.0002 μg/mL TgNTPase or NcNTPase and was incubated at 37 °C for 10 min. After incubation, 2.5 μL 0.1N HCl was added. Then, 12.5 μL of 2× biofluorescent reaction solution was added and measured by PHERAstar FS. 

### 4.6. Statistics

Values are expressed as the mean ± standard error of the mean (SEM) from at indicated replicate samples in each experimental group; experiments were replicated to ensure consistency. Statistical significance was determined using Student’s *t*-test. Values as * was considered to be statistically significant if their *P* values were *P* < 0.05. Signal-to-Background ratio (S/B), Signal-to-Noise ratio (S/N) and Z’-factor were calculated by formula as described in our previous report [21]. In addition to that, general criterion sufficient to do HTS was validated according to previous report [22].

## Figures and Tables

**Table 1:** Qualities of independent experiments.

Plate	Enzyme	S/B	S/N	Z’-Factor	Measurement	Number of Sample
96 well	NcNTPase	4.13	3.91	0.074	Absorbance	*n* = 3
	TgNTPase	7.71	38.4	0.077	Absorbance	*n* = 3
	NcNTPase	7.60	234.9	0.960	Fluorescence	*n* = 3
	TgNTPase	11.28	342.4	0.876	Fluorescence	*n* = 3
384 well	NcNTPase	15.3	551.3	0.970	Fluorescence	*n* = 16
	NcNTPase	12.7	344.3	0.970	Fluorescence	*n* = 16
	TgNTPase	8.44	500.2	0.925	Fluorescence	*n* = 16
	TgNTPase	8.28	394.1	0.890	Fluorescence	*n* = 16

## Supplementary Materials

The following are available online at https://www.mdpi.com/1660-3397/18/3/161/s1, Supplementary figure: Schematic figure indicates procedures and reagents of the novel fluorescent assay using robot arm.
