# Supplementary material for: Establishment of Novel High-Standard Chemiluminescent Assay for NTPase in Two Protozoans and Its High-Throughput Screening
Source: Mar Drugs. 2020 Mar 13;18(3):161. doi: 10.3390/md18030161 (PMC7142789; doi:10.3390/md18030161)
Supplement: Supplementary file 1 [file marinedrugs-18-00161-s001.pdf]

Supplementary figure.

Schematic figure indicates procedures and reagents of the novel fluorescent assay using robot arm.

Enzyme reaction sol.
50 mM HEPES-KOH pH 7.5
1 mM ATP
6 mM $Mg(CH_3COO)_2$
N.c. or T.g. NTPase

↓ Put reagent by robot arm

Incubation at 37℃ for 10 min
and then, added 0.1N HCl

↓ Put reagent by robot arm

2x Biofluorescent reaction sol.
100 mM Tris HCl (pH7.5)
10 mM $MgCl_2$
2 mM Glucose
2 U/ml ADPHK
2 U/ml G6PDH
2 U/ml Diaphorase
200 μM NADPH
100 μM Resazurin
0.01 % BSA
0.05 % Triton X-100
20 mM N-ethylmaleimide

↓

Measured fluorescent
intensity at ex. 540/em. 590
